# Epigenetics and Sex-Specific Fitness: An Experimental Test Using Male-Limited Evolution in *Drosophila melanogaster*

**DOI:** 10.1371/journal.pone.0070493

**Published:** 2013-07-29

**Authors:** Jessica K. Abbott, Paolo Innocenti, Adam K. Chippindale, Edward H. Morrow

**Affiliations:** 1 Department of Biology, Section for Evolutionary Ecology, Lund University, Lund, Sweden; 2 Department of Animal Ecology, Evolutionary Biology Centre, Uppsala University, Uppsala, Sweden; 3 Biology Department, Queen’s University, Kingston, Ontario, Canada; 4 School of Life Sciences, University of Sussex, Brighton, United Kingdom; North Carolina State University, United States of America

## Abstract

When males and females have different fitness optima for the same trait but share loci, intralocus sexual conflict is likely to occur. Epigenetic mechanisms such as genomic imprinting (in which expression is altered according to parent-of-origin) and sex-specific maternal effects have been suggested as ways by which this conflict can be resolved. However these ideas have not yet been empirically tested. We designed an experimental evolution protocol in *Drosophila melanogaster* that enabled us to look for epigenetic effects on the X-chromosome–a hotspot for sexually antagonistic loci. We used special compound-X females to enforce father-to-son transmission of the X-chromosome for many generations, and compared fitness and gene expression levels between Control males, males with a Control X-chromosome that had undergone one generation of father-son transmission, and males with an X-chromosome that had undergone many generations of father-son transmission. Fitness differences were dramatic, with experimentally-evolved males approximately 20% greater than controls, and with males inheriting a non-evolved X from their father about 20% lower than controls. These data are consistent with both strong intralocus sexual conflict and misimprinting of the X-chromosome under paternal inheritance. However, expression differences suggested that reduced fitness under paternal X inheritance was largely due to deleterious maternal effects. Our data confirm the sexually-antagonistic nature of Drosophila’s X-chromosome and suggest that the response to male-limited X-chromosome evolution entails compensatory evolution for maternal effects, and perhaps modification of other epigenetic effects via coevolution of the sex chromosomes.

## Introduction

The ubiquity of sexual dimorphism reveals that divergent selection on males versus females is very widespread. However, because most of the genome is shared between the sexes, intersexual genetic correlations can potentially impose constraints on the evolution of dimorphism. Sexually antagonistic alleles are those that are favoured in one sex but cause the other to depart from its sex-specific optimal phenotype, a phenomenon which is known as intralocus sexual conflict [1]. Evidence that intralocus sexual conflict is both common and consequential has accumulated in recent years [2]–[5]. For example, one recent study found that increased testosterone level was favoured in male voles but selected against in female voles, resulting in a negative genetic correlation between the fitness of male and female relatives [6]. Sexually antagonistic selection on expression levels, recently inferred to be widespread in the *Drosophila melanogaster* genome, may be an important mediator of intralocus sexual conflict [7].

Because intralocus sexual conflict results in the displacement of one or both sexes from their phenotypic optimum, it reduces fitness, a phenomenon known as gender load [8], [9]. Selection should therefore work to resolve intralocus sexual conflict and reduce gender load, for example by increasing the degree of sexual dimorphism and thereby bringing each sex closer to its fitness optimum. Several mechanisms by which selection can reduce and eventually eliminate intralocus sexual conflict are recognized, including the evolution of sex-specific modifiers, gene duplication and subsequent divergence, relocation of sexually antagonistic genes to the sex chromosomes, sex-specific maternal effects, and genomic imprinting [3], [10], [11]. Here we will focus on sex-specific maternal effects and genomic imprinting.

Maternal effects could contribute to the resolution of intralocus sexual conflict in at least two ways. Firstly, females could mitigate the negative effects of intralocus sexual conflict in their offspring by sex-specifically provisioning eggs with transcriptional factors that affect gene expression. Secondly, females could invest more in their offspring of a specific sex to reduce intralocus sexual conflict. This could be accomplished either by differential energetic investment in individual eggs or offspring, or by biasing the sex ratio based upon mate quality; when mated to a high quality male a female should produce male-biased broods, but when mated to a low-quality male a female should produce female-biased broods. To date there are however relatively few examples of these processes. Maternal (and paternal) effects are known to influence offspring fitness in *Drosophila* [12], [13], and egg provisioning is a possible (but as yet unconfirmed) explanation for this observation [14]. Selective investment in offspring to reduce intralocus sexual conflict has been shown in lizards [15], [16], red deer [17], barn owls [18], flour beetles [19], and *Drosophila* [20], [21]. Adaptive maternal effects therefore clearly have the potential to mitigate intralocus sexual conflict, but their relative importance is unknown.

Genomic imprinting occurs when gene expression levels are modified according to parent-of-origin [22]. The most common explanation for this phenomenon (although not the only one, e.g. [23]) is the parental conflict hypothesis [24], which states that because of an imbalance in the investment in offspring, paternally inherited genes should try to maximize maternal investment in the current reproductive episode, while maternally inherited genes should try to equalize investment across all reproductive episodes, all else being equal [25]–[27]. However genomic imprinting could also contribute to the resolution of intralocus sexual conflict if parents can imprint genes according to offspring sex [28]. For example, a successful male should have an excess of alleles that are beneficial to males but detrimental to females. Since it is the sperm genotype that determines offspring sex in XY systems, males should therefore imprint their genome such that male-benefit alleles at sexually antagonistic autosomal or X-linked loci should be turned “on” in Y-bearing sperm, but turned “off” in X-bearing sperm. Although there has been some theoretical treatment of this issue to date [28], [29], there is relatively little empirical data on the contribution of imprinting to the resolution of intralocus sexual conflict. The fruit fly *Drosophila melanogaster* is known both to experience substantial intralocus sexual conflict (at least in some laboratory populations) [1], [7], [8], [30], [31] and to exhibit genomic imprinting at certain loci [32]–[36]. However the contribution of genomic imprinting to the resolution of intralocus sexual conflict has, to our knowledge, never been empirically tested.

The imprinting mechanism found in *D. melanogaster* has little in common with that found in plants and mammals. Plants and mammals imprint genes via selective methylation of specific loci, resulting in decreased expression of the methylated genes [26]. In contrast, *Drosophila* imprint genes based on their proximity to heterochromatic chromosomal regions (i.e. regions with densely packed DNA [32]). This is also known as position-effect variegation, and has mainly been investigated by translocating phenotypic markers to locations in or near heterochromatic regions, and then measuring parent-of-origin effects on phenotypic expression [34], [37]. It has generally been assumed that imprinting in *Drosophila* is of no adaptive significance, since this would require non-random location of imprinted genes relative to heterochromatic regions [32]. Evolution of sex-specific expression via chromosomal relocation is however a well-known phenomenon [11], [38], [39]. This suggests that imprinting of sexually antagonistic loci via their relocation close to or away from heterochromatic regions should theoretically be possible. It is also likely that populations will harbor standing genetic variation in imprinting patterns, since new evidence suggests that variation in imprinting patterns can arise via mutation in as little as 550 generations in *Drosophila* [40]. In addition, artificial insertion of phenotypic markers of large effect may not be particularly informative about the importance of imprinting to naturally-occurring phenotypic variation, since the direction and magnitude of the effect is marker-dependent, even for insertions into the same chromosomal region [34].

The X-chromosome determines sex in *Drosophila* (via the ratio of X-chromosomes to autosomes) and is known to be enriched for sexually antagonistic genetic variation [7], [38], [39], [41], so it seems a likely candidate for genomic imprinting. We developed an experimental evolution protocol to investigate the contribution of sexually antagonistic genetic variation on the X-chromosome to male fitness in *Drosophila*. We employed male-limited X-chromosome (MLX) evolution, in which a chromosomal aberration in females enforces father-son transmission of X-chromosomes (see Methods), reversing the normal pattern of inheritance for the sex chromosomes. Two main objectives with this experimental protocol were to quantify the relative contribution of the X-chromosome to the increase in male fitness seen in a previous whole-genome male-limited evolution experiment [8], [30], and to identify which genes changed in expression level as a result of the MLX evolution. A presentation of these results, which will deal with sexually antagonistic effects in individuals of both sexes, is currently in preparation. A third important objective (and our focus in this paper) was to determine whether epigenetic effects on the X-chromosome affect male fitness in this species, and whether these epigenetic effects can evolve on short time scales. Although we were specifically interested in imprinting effects, our data was also suitable for investigating the influence of maternal effects on male fitness, allowing us the opportunity to study the importance of several types of epigenetic effect.

If imprinting of the X occurs, we predict that father-son X-transmission will be detrimental to sons, and selection will operate against such epigenetic feminization. Similarly, if adaptive evolution of maternal effects is important in the resolution of intralocus sexual conflict, then we expect that male-benefit maternal effects should be enhanced and male-detriment maternal effects suppressed in our experimental populations. We found that one generation of father-son transmission of the X-chromosome resulted in markedly decreased male fitness, which was more than recovered after 40 generations of adaptation to father-son transmission; by this point, MLX-evolved males had substantially higher fitness than control males, even those with conventional (maternal) inheritance of their X-chromosome. These results are consistent with the evolution of epigenetic modifiers, X-chromosome adaptation to maternal effects, and the existence of strong X-linked sexual antagonism.

## Methods

See Table 1 for list of abbreviations.

**Table 1:** List of abbreviations used in this paper.

Abbreviation	Description
C	Control treatment
CDX	Treatment to detect epigenetic effects; males with a Control X-chromosome that have been produced by DX females
DX	Females with a double X-chromosome (i.e. two X-chromosomes attached at the centromere)
GO	Gene Ontology database
LHm	The outbred laboratory stock used in these experiments
MLX	Male-limited X-chromosome evolution treatment (and males derived from this treatment)
RB	Recombination box; accessory population to MLX populations allowing recombination between X-chromosomes
SA	Sexually antagonistic, here used in the context of sexually antagonistic zygotic drive

### Fly Stocks

All populations were derived from the LHm stock [42], and were maintained using the same general culturing protocol. LHm flies are maintained at 25°C on a 12–12 light-dark cycle, at 50% relative humidity, and fed with cornmeal-molasses-yeast medium. Eggs are laid on day 1, adult flies eclose on day 9 or 10, are mixed between vials on day 12, and 16 pairs per vial are randomly selected to produce the next generation. Males and females interact for 48 hours in vials supplemented with 6 mg of live yeast, and then are transferred to new (yeastless) vials for oviposition; females are given an 18 hour window in which to lay eggs. At the end of this period all adult flies are discarded (new day 1). The 48 hour period with controlled density of 16 pairs ensures that all adult flies experience the same conditions while competing for matings (males) or yeast (females). Once egg laying is completed, vials are trimmed to 150±10 eggs in order to keep larval densities constant. A total of 56 vials are maintained (1 792 breeding adults and about 8 000 juveniles).

### MLX Experimental Protocol

**Figure 1:**
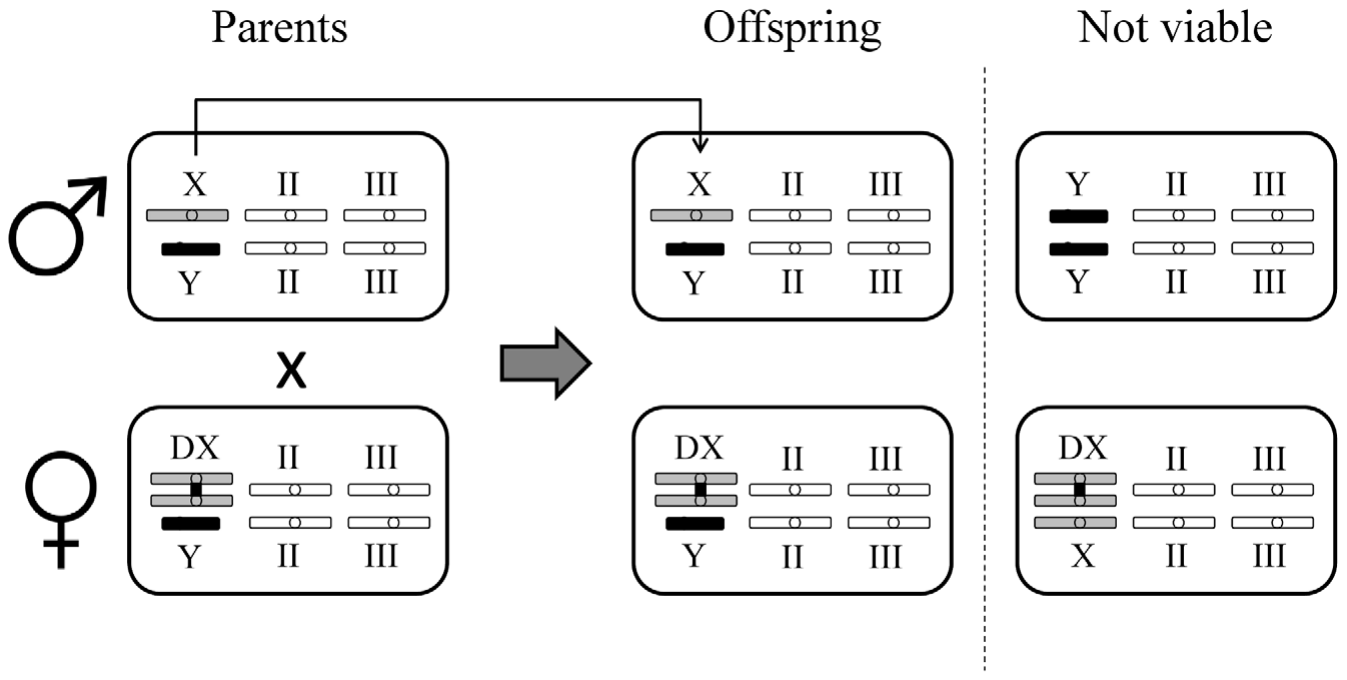
MLX evolution protocol. Males are mated to females with a double X-chromosome (DX), which forces father-son transmission of the X-chromosome, and produces wildtype males with a paternally inherited X-chromosome and a maternally inherited Y-chromosome. New DX females with a paternally inherited Y-chromosome are also produced. Triple-X and double-Y individuals are not viable.

Male-limited X-chromosome evolution is made possible in *D. melanogaster* by the use of compound X (i.e. “double X” or DX) females, which have two X-chromosomes that are linked at the centromere (*C(1)DX, y, f*), and carry a random Y-chromosome (and autosomes) from the LHm population. When wildtype males mate with females of this karyotype, X-bearing sperm fertilize Y-bearing eggs, creating father-son transmission of the X, but leaving transmission of the autosomes unaffected (Figure 1). Prior to beginning the experiment, the population of DX females used in setting up the experimental populations was backcrossed to the LHm base stock for 6 generations (enough to ensure that their autosomal genetic material was >98% identical to the base stock). Three replicate MLX populations were each started by taking 480 males from the LHm stock population, and then mating them to an equal number of backcrossed DX females (total population size = 960). Populations were subsequently reduced to a total population size of 640 for logistical reasons, and kept in that state until the end of the experiment. Closed replicate Control (C) populations of the same population size were also created and maintained alongside the MLX populations. Since all populations were started simultaneously, populations within the same treatment should be no more or less related to each other than populations from different treatments.

In order to prevent hitchhiking of deleterious alleles via clonal selection, we also simultaneously started a “recombination box” treatment for each replicate MLX population [8]. Each recombination box (RB) initially included 48 LHm individuals of each sex (i.e. 10% of the MLX population size) which is enough to override any effects of genetic hitchhiking of deleterious alleles [43]. The RB population size was later reduced to 32 individuals of each sex, in order to keep it proportional to the reduced main population size. In each generation 32 new males for the RB were randomly selected from the MLX experimental populations and combined with 32 (non-virgin) RB females on day 12, while 32 RB males were added back to the main MLX population. This ensures a constant inflow of new X-chromosomes to the recombination box, and outflow of recombined X-chromosomes back into the main MLX populations (Figure S1 in File S1).

### Fitness Protocols

After 40 generations of experimental evolution, we measured male fitness using a standard eye-marker protocol (e.g. [30]). A vigorous LHm-derived population with a recessive brown eye-colour marker (*bw^1^*) is used as competitors to the target (red-eyed) populations. On day 12 from egg, 4 target males are combined with 12 brown-eyed males and 16 (non-virgin) brown-eyed females. They are allowed to interact for 48 hours, during which the target males compete for matings with the brown-eyed males. Females are then transferred to individual test tubes for 18 hours to lay eggs, after which they are discarded. Male fitness is measured as the proportion of adult offspring sired when in competition for matings with the competitor males, and because target males within the same vial are not independent, one vial represents one sample. Fitness was compared between three groups: Control (C) males, MLX males, and males that were the offspring of a Control male and a DX female (CDX; see Figure S2 in File S1). The aim of this design was to test whether father-son transmission of the X results in a reduction in male fitness, and whether this reduction can be recovered as a result of MLX evolution. In order to avoid pseudoreplication, statistical analysis (one-way anova in R [44]) was carried out using population means, which were based on a sample size of 20 vials each. We also tested for offspring sex ratio differences by mating males of all three treatments to Control females and recording the adult offspring sex ratios (see Figure S2 in file S1). Statistical analysis was carried out in the same way as for fitness, except that the sex ratio values were first arcsine square-root transformed.

### Gene Expression Analysis

Gene expression data was collected after 50 generations of experimental evolution. RNA for gene expression analysis was extracted using Trizol (Invitrogen) and purified with an RNeasy Mini Kit (Qiagen). Sample size was 6 independent groups of 8 male flies per treatment; these 6 groups represent 2 replicates from each of the three replicate experimental populations (for a total of 18 arrays and 144 flies). RNA quantity and quality was assessed using an Agilent Bioanalyzer (Agilent Technologies) prior to sample preparation and hybridisation at the Uppsala Array Platform (following the manufacturer's instructions for GeneChip Drosophila Genome 2.0 Affymetrix microarrays).

**Figure 2:**
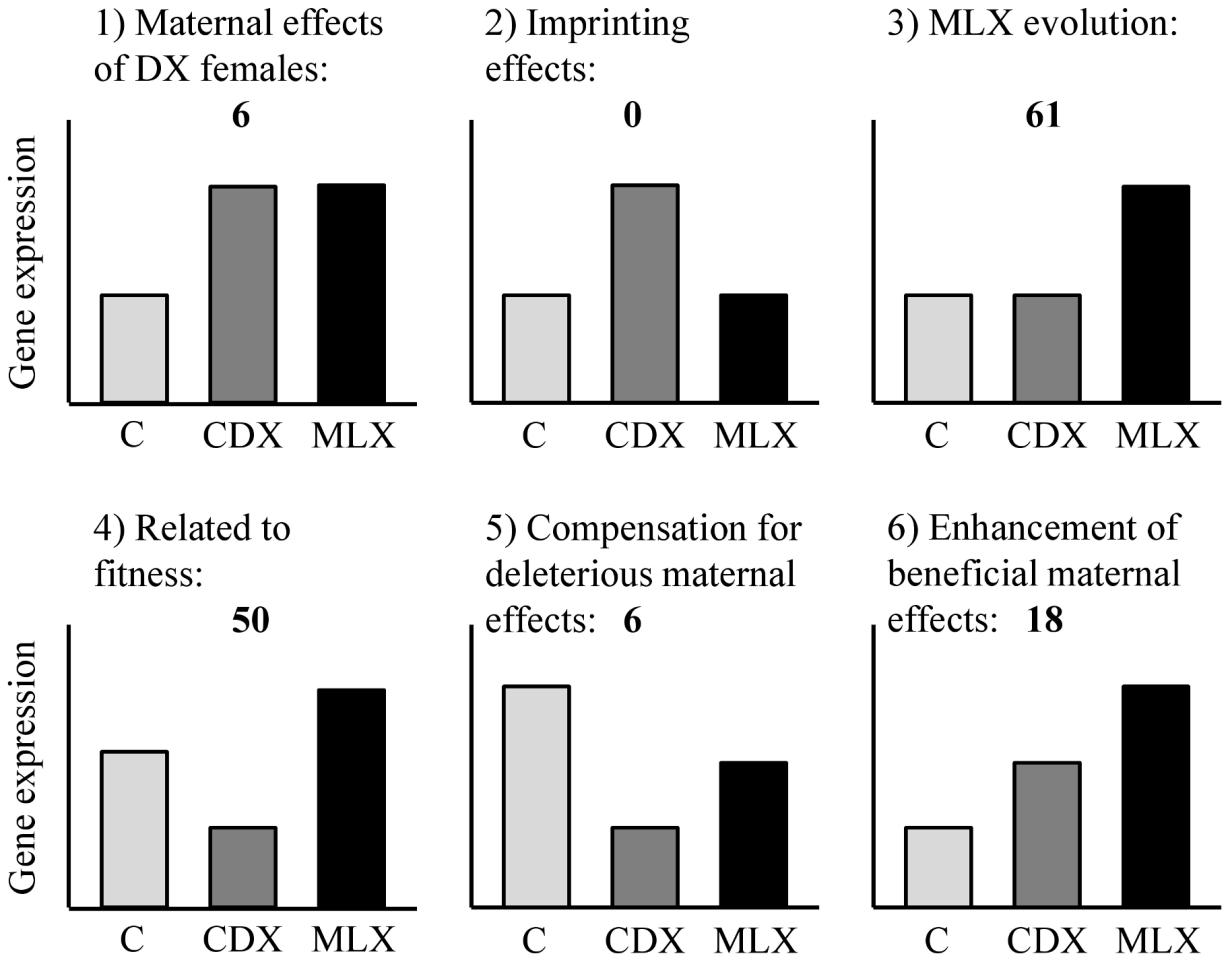
Graphical representation of transcript categories, and numbers of transcripts found in each category. Compare category 4 with Figure 3 (fitness results) and Figure S3 in file S1 (sex ratio results).

Analysis of gene expression data was carried out in BioConductor 2.4 (http://www.bioconductor.org). Microarray data were pre-processed using Robust Multichip Average (RMA) in the “affy” package [45]. Significant differences in gene expression levels between treatments were tested using a model that included Treatment as a fixed factor and Population as a random factor nested within Treatment, with a false discovery rate of 0.05. Transcripts that differed in expression between treatments could be classified into 6 different categories (see Figure 2 for a graphical representation):

1. Transcripts that are up- or down-regulated in CDX and MLX relative to Controls. This should represent maternal effects of the DX females, since CDX and MLX males both come from DX mothers.
2. Transcripts that are up- or down-regulated in CDX but with no difference between Control and MLX. This should presumably represent an effect of feminization of the X via imprinting. Since the X-chromosome is usually inherited from father to daughter, fathers should imprint their X-chromosome to increase female fitness. CDX males are therefore expected to have feminized X-chromosomes since there has been no opportunity to adapt to father-son transmission of the X-chromosome in this treatment. In contrast, MLX males have had the chance to adapt to father-son transmission of the X-chromosome and are therefore expected to have evolved normal expression levels for most transcripts, either via compensatory evolution or via modification of the imprint. Control males have normal transmission of the sex chromosomes, so they should have normal expression levels. Because the fitness-related transcripts (category 4, see below) could potentially also include some imprinted genes, the number of transcripts assigned to the imprinting category should be considered a lower bound for the total number of X-linked imprinted genes.
3. Transcripts that are up- or down-regulated in MLX but with no difference between Control and CDX. This should represent the changes that have occurred as a result of MLX evolution.
4. Transcripts with up- or down-regulation in the order CDX-C-MLX should represent fitness-related transcripts. For this group it is not possible to determine whether this pattern is due to evolution of the imprint, or due to detrimental maternal effects in the CDX males which are then cancelled out in MLX males via compensatory evolution.
5. Transcripts with up- or down-regulation in the order CDX-MLX-C should represent deleterious maternal effects of the DX females that are accommodated or reduced via adaptation to the maternal genetic environment. This category could potentially also include deleterious effects that are a result of maternal imprinting.
6. Transcripts with up- or down-regulation in the order C-CDX-MLX should represent beneficial maternal effects of the DX females that are enhanced via adaptation to the maternal genetic environment. This category could potentially also include beneficial effects that are a result of maternal imprinting.

Not all categories are unambiguous, so a Venn diagram showing the pattern of significant pairwise comparisons between the three treatments and their relationship to the six categories discussed here can be found in the (Figure S3 in file S1).

The Gene Ontology database (GO) includes data on gene function, arranged into hierarchical categories, so transcripts from sufficiently numerous categories were tested for overrepresentation of GO categories. We also tested chromosomal distribution, tissue-specificity, and association with sex-specific fitness and sexual antagonism, as measured in a previous study of the LHm population [7]. Overrepresentation of GO categories was analysed using hypergeometric testing (“hyperGTest” in R). Chromosomal distribution was tested using a χ^2^ test (“chisq.test” in R). Tissue specificity was measured in the same manner as in a previous expression analysis of the LHm population [7]. Briefly, tissue-specific transcripts (i.e. with two-fold higher expression than in the whole fly) were identified from the Gene Expression Omnibus, accession number GSE7763, and then overabundance of genes of interest in a target tissue was determined by carrying out a one-tailed Fisher's exact test (“fisher.test” in R) on the observed and expected tissue-specific genes of interest, compared to the overall number of tissue-specific genes in each tissue. Significance values were Bonferroni-corrected for testing on multiple tissues (*n* = 17). Association with sex-specific fitness and sexual antagonism was analysed using two-tailed mean-rank gene set enrichment (MR-GSE) tests. For all transcripts in [7], the association between expression level and sex-specific fitness was measured using a regression model. Those transcripts with a significant interaction term were additionally classified as sexually antagonistic (i.e. there is overlap between the sex-specific fitness classes and the sexually antagonistic class). The MR-GSE test uses the previously acquired data to rank all transcripts in terms of their association with male fitness, female fitness, or sexual antagonism, and then determines where our sets of significant transcripts fall in relation to this ranking. If our set of transcripts has a higher mean ranking in e.g. the male fitness list than would be expected by chance, this is evidence of a significant association.

If the X-chromosome can be imprinted, paternal X-chromosomes destined to reside in daughters should be modified to have feminized expression levels, as discussed above. We therefore also tested whether X-linked transcripts tended to be feminized in CDX males using a χ^2^ test (“chisq.test” in R). That is, whether the difference in expression between CDX males and Control males was in the same direction as the difference in expression between females and males more often than expected by chance. We did not include the MLX males in this analysis because it is only possible to directly identify imprinting effects in the CDX group. The CDX males have Control-derived X-chromosomes, and can therefore only have X-linked expression levels which differ from the Controls as a result of epigenetic effects. In contrast, the MLX males have X-chromosomes that have undergone evolution and these X-chromosomes are likely to be different from the Control X-chromosomes. This means that for the MLX males, we cannot distinguish between expression differences as a result of evolution of the imprint, and those resulting from recombination and fixation of sexually antagonistic loci. For the feminization analysis we considered all X-linked transcripts in order to see if there was any overall pattern, apart from the effects of individually significant transcripts. For the sake of comparison, the same analysis was carried out for all autosomal transcripts as well.

## Results

**Figure 3:**
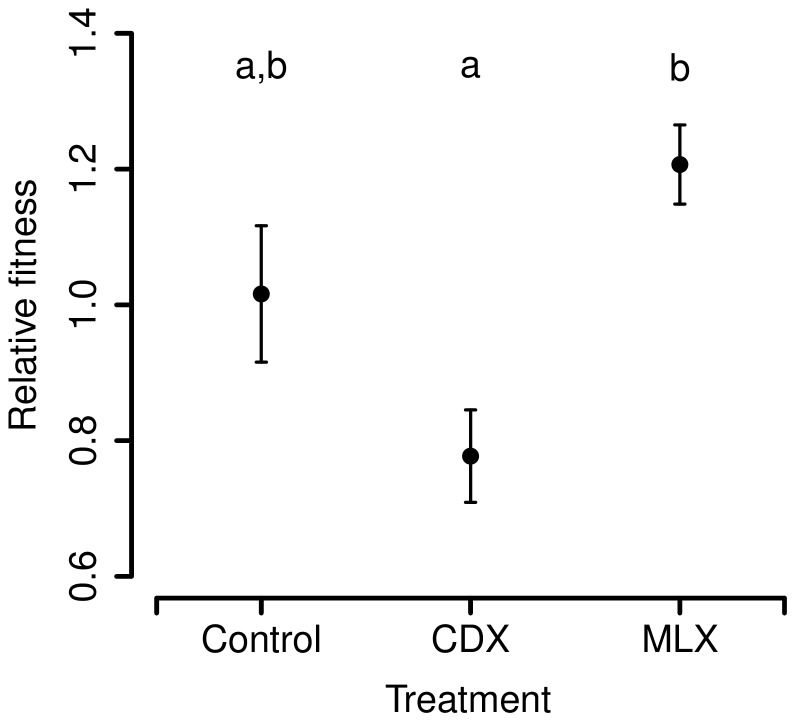
Fitness differences between the treatments. MLX males have higher fitness than CDX males (i.e. males with a paternally transmitted X-chromosome, produced by crossing a Control male to a DX female). Fitness was measured as the proportion of adult offspring sired when in competition for matings with marked competitor males. Error bars denote SE.

We expected that CDX males would have decreased fitness due to epigenetic feminization of the X-chromosome and/or deleterious maternal effects, but that this effect would be cancelled out in MLX males as a result of male-limited evolution. We therefore predicted that MLX males would have the highest fitness, Control males have intermediate fitness, and CDX males would have the lowest fitness. Our results were consistent with this prediction (F_2, 6_ = 7.666, *P* = 0.0223; Figure 3). There was also a similar effect of treatment on sex ratio (F_2, 6_ = 9.757, *P* = 0.013; Figure S4 in file S1), such that MLX males produced significantly more surviving adult male offspring than CDX males did. Note that this pattern cannot be a result of direct effects of the X-chromosome since the sons have a maternally inherited Control X-chromosome (Figure S2 in file S1), so it suggests the existence of other epigenetic effects on fitness.

Although the fitness results were consistent with a potential imprinting effect, we needed to investigate differences in patterns of gene expression between the experimental treatments (Figure 2) in order to be able to suggest possible mechanisms. A total of 141 transcripts were found to differ between at least two of the treatments (Figure S3 in file S1). CDX males had no transcripts with a pattern of expression that was unique to the treatment (i.e. category 2 in Figure 2), suggesting that imprinting is not likely to be responsible for the decrease in fitness in this group. A likely alternative explanation is maternal effects, since a total of 30 transcripts were associated with various types of maternal effects (categories 1, 5 and 6 in Figure 2, see also Figure S3 in file S1). The categories with the highest number of transcripts that had changed in expression were those associated with MLX evolution and with fitness (categories 3 and 4 in Figure 2), and so further results are reported from these categories only.

Overrepresented Gene Ontology (GO) terms for “MLX evolution” transcripts (category 3) included muscle fibre assembly (in biological processes), myofibril, contractile fibre, and extracellular region (in cellular components), and oxidoreductase activity (in molecular functions), which suggests that there has been a change in muscle function and activity in MLX males. A full list of GO terms can be found in Table S1 in File S1. There was no evidence of any skew in chromosomal distribution among these transcripts (χ^2^ = 4.7796, df = 3, *P* = 0.1887). “MLX evolution” transcripts were associated with tissue-specific expression in the ejaculatory duct, head, heart, and carcass. They were also significantly positively associated with male fitness (*P* = 0.0116) and sexual antagonism (*P* = 0.0390), but did not show any relationship with female fitness (*P*>0.3).

Overrepresented GO terms for “fitness” transcripts (category 4) included ATP-dependent proteolysis (in biological processes), membrane (in cellular components), and peptide hydrolase activity (in molecular functions), which may represent a change in metabolic activity in MLX males. A full list of GO terms can be found in Table S2 in File S1. There was evidence of overrepresentation of genes located on chromosome 4 among these transcripts (χ^2^ = 25.830, df = 3, *P* = 1.035*10^−5^). However chromosome 4 is very gene-poor, so this effect is only a matter of 4 observed genes relative to 0.3 expected genes and is therefore not very informative. There was no evidence of skew in chromosomal distribution when chromosome 4 was excluded (χ^2^ = 1.852, df = 3, *P*>0.3). “Fitness” transcripts showed a similar pattern of tissue-specific expression to “MLX transcripts”, and were associated with the accessory gland, ejaculatory duct, head, and carcass. As with “MLX evolution” transcripts, “fitness” transcripts were significantly positively associated with male fitness (*P* = 0.0027) and sexual antagonism (*P* = 0.0056), but did not show any relationship with female fitness (*P*>0.3).

Although the results of the test for direction of change in expression of X-linked transcripts in CDX males compared to Control males was highly significant (χ^2^ = 82.7517, df = 3, *P*-value <2.2*10^−16^), there was no evidence that these transcripts had feminized expression levels in CDX males (Table 2). Instead the data suggest that CDX males had higher expression of X-linked transcripts overall, regardless of whether those transcripts are male-biased or female-biased in the Control population. Autosomal transcripts showed no sign of feminization of expression in CDX males either (Table S3 in File S1).

**Table 2:** Results of test for feminization of X-linked transcripts in CDX males.

	Up-regulated in females		Down-regulated in females	
	Up-regulated in CDX	Down-regulated in CDX	Up-regulated in CDX	Down-regulated in CDX
**Observed**	116.00	69.00	187.00	71.00
**Expected**	110.75	110.75	110.75	110.75

If CDX males (i.e. males with a paternally transmitted X-chromosome, produced by crossing a Control male to a DX female) have feminized X-chromosomes due to imprinting, then the change in expression of X-linked transcripts relative to Control males should be in the same direction as extant sexual dimorphism more often than expected by chance (first and last columns). Rather than being consistent with feminization, the data suggest increased expression of many X-linked transcripts in CDX males, regardless of whether these transcripts are usually male-biased or female-biased (first and third columns). This result is consistent with coevolution between the sex chromosomes (see main text). Note that the total number of transcripts in the analysis is less than the total number of X-linked genes because uninformative transcripts (i.e. those without gene annotation information, or those whose expression was the same across all samples) were filtered out during pre-processing.

## Discussion

Males expressing X-chromosomes that had undergone just one generation of father-son transmission displayed reduced fitness, while those inheriting X-chromosomes that had undergone many generations of MLX evolution showed increased fitness (Figure 3). The difference in fitness between these treatments (approximately 40%) is substantial. The improvement in fitness of MLX relative to control males is consistent with the presence of X-linked polymorphism for sexually-antagonistic alleles in the LHm (base) population. Indeed, the degree of increase in fitness is similar to that seen in the whole-genome male-limited evolution experiments of Rice [43], [46] and Prasad *et al.* [8], despite the fact that only the X-chromosome was limited to males in this experiment. Although a point estimate, it also corroborates the findings of Gibson *et al.* [41] for the predominance of X-linked sexually antagonistic effects in this species. However in contrast to our expectation, expression data suggested that the initial decline in fitness from patrilineal X-chromosome transmission was not related to maladaptive X-linked imprinting effects which were then reversed by adaptation to father-son transmission. This is evidence that resolution of intralocus sexual conflict on short time scales is unlikely to be achieved via genomic imprinting in *Drosophila*. Instead, these data point to deleterious maternal effects that were then cancelled out by MLX evolution (Figure 2). Here we discuss several ways in which epigenetic phenomena could have affected the observed results.

The DX females essential to the MLX evolution protocol carry several phenotypic markers and are less vigorous than wildtype females (J. Abbott, personal observation), so it is unsurprising that this could result in carry-over of deleterious effects to their offspring [47]. There were relatively few transcripts in the simple maternal effects category (Category 1 in Figure 2), which is rather unexpected given the measureable fitness decrease in CDX males. However maternal effects are usually expected to attenuate over time [48], so it could be the case that DX females’ maternal effects mainly influence expression differences in the embryo or larval stages, which we did not measure here. Poor juvenile condition of the sons of DX females would then carry over to the adult stage and cause a reduction in male fitness, without necessarily resulting in substantial difference in expression profiles in the adult male flies. The significant effects seen here of DX females on offspring gene expression also have wider implications. Attached-X genetic constructs are a commonly-used tool in *Drosophila* (e.g. [12], [40], [49]), and these results suggest that such constructs may have larger transgenerational phenotypic effects than has previously been appreciated. As long as the DX construct has consistent effects across all genotypes then qualitative conclusions will be unaffected, but if the effect of the DX varies according to genetic background then is this cause for concern. Unfortunately this is currently unknown, so a formal investigation of the effect of the DX across various genetic backgrounds would be of value. Even for other model organisms more caution is probably warranted when using non-wildtype genetic constructs, rather than simply assuming that such effects will be negligible.

It’s also possible that most deleterious maternal effects were subsequently ameliorated via compensatory evolution in the MLX populations, leading them to end up among the “fitness” transcripts (Category 4 in Figure 2). A more thorough treatment is currently in preparation, where we will discuss male-limited X-chromosome evolution effects in individuals of both sexes that are not subject to deleterious maternal effects of DX females, so we will only briefly deal with this issue here. Previous male-limited (ML) experimental evolution studies across the whole genome found increased male fitness as a result of this ML evolution (reviewed in [49]). MLX evolution is therefore expected to result in increased male fitness–even in the absence of imprinting–via fixation of X-linked male-benefit/female-detriment alleles that normally experience counter-selection in females. Our results are remarkably consistent with this prediction, as we found an increase in fitness in MLX males relative to Control males which seemed to be mediated by an increase in the expression of male-benefit and sexually antagonistic (male-benefit/female-detriment) genes, particularly those associated with metabolism. Because of this, we cannot rule out the possibility that the decrease in fitness seen in CDX males is due to X-linked imprinting, while part of the increase in fitness seen in MLX males is due to MLX evolution which compensates for the maladaptive imprint. However three lines of evidence speak against interpreting the decrease in fitness seen in CDX males as a result of X-chromosome imprinting. Firstly, there were no transcripts that were unambiguously indicative of imprinting effects (Category 2 in Figure 2). Secondly, there was no evidence for feminization of expression patterns of paternally inherited X-chromosomes in CDX (unevolved) males (Table 2). Finally, the proportion adult male offspring of the experimental males (i.e. the Control, CDX, and MLX males used as fathers in the sex ratio assay; Figure S4 in file S1) followed the same pattern as for fitness (Figure 3), yet this pattern cannot be explained by imprinting of the X-chromosome. This is because the male offspring had Control X-chromosomes which they inherited from the Control mothers that were used in the sex ratio assay (see Methods and Figure S2 in file S1). We therefore consider it unlikely that the decrease in fitness in CDX males is due to mis-imprinting of the paternal X-chromosome. This is consistent with previous results which also suggest that although maternal and paternal effects can influence expression levels in *Drosophila* [13], this was in once case determined to be unlikely to be due to imprinting [14]. Although the mechanism in [13] is unknown, one possibility is changes in abundance of transcription factors [14].

There are three other potential explanations for low fitness of CDX males which all relate to the maternal transmission of the Y-chromosome rather than paternal transmission of the X-chromosome: (i) deleterious effects of recombination between the X and the Y as a result of their passage through the DX females, (ii) maternal imprinting of the Y-chromosome, and (iii) sexually antagonistic (SA) zygotic drive. Although it is currently not known whether the X and Y can recombine in females, there is evidence that DX females can imprint the Y-chromosome [50]. If maternally inherited Y-chromosomes are imprinted differently than paternally inherited ones, this could explain the reduced fitness seen in CDX males since the Y-chromosome has extensive regulatory functions [51] and has recently been shown to influence genome-wide imprinting effects [40]. In SA zygotic drive, the sex chromosomes attempt to bias their transmission by harming offspring not carrying the respective chromosome [52]. For example, the compound X-chromosome carried by the DX females is female-limited and could accumulate son-harming genetic variation, resulting in a female-biased sex ratio or reduced fitness of sons. Some evidence for this kind of effect has been presented and interpreted as resulting from imprinting of the Y-chromosome by the X in paternal transmission [12], [36]. Unfortunately we cannot test the latter two hypotheses directly since the Affymetrix chip only contains a handful of Y-linked transcripts, too few for statistical testing, and it is unknown which genes might be associated with SA zygotic drive.

A final intriguing possibility is that at least part of the adaptation resulting from male-limited X-chromosome selection has come about through a coevolutionary interplay between the X- and Y-chromosomes. While long characterized as a minor genomic player–non-recombining, degenerate and with a mere handful of functional loci–recent evidence suggests a major role for the Y-chromosome in determining male fitness. The *D. melanogaster* Y-chromosome is now known to produce genome-wide regulatory effects [50] which have been shown to undergo diversification in isolated laboratory lines derived from a single founder male within several hundred generations [40]. The latter study found marked differences in the modulation of gene expression via altered chromatin states, which was achieved by X-linked rDNA-silencing mediated by the Y-chromosome. The LHm population used here has previously been shown to be polymorphic for Y-chromosomes, with epistatic interactions with other chromosomes having a large impact on male fitness [42]. These observations make it plausible that Y-chromosome composition evolved under male-limited X-chromosome evolution, presumably to optimize male fitness in concert with the evolving X-chromosome population. Coevolution of the X- and Y-chromosomes during male-limited X-chromosome evolution could also explain our sex ratio results (Figure S4 in file S1), where male offspring survival seemed to be determined by the match between the paternal sex chromosomes (Figure S2 in file S1).

The fitness effect of individual Y-chromosomes has been found to be highly non-additive and dependent on the specific genetic background in the LHm population, so it was previously assumed that Y-chromosome polymorphism was maintained mostly via epistasis, non-transitivity, and stochastic effects [42]. However since the evolved X-chromosomes in this study are likely to be enriched for male-benefit sexually antagonistic alleles, our results suggest that Y-chromosome polymorphism in the LHm stock could instead be maintained by sexual antagonism on the X-chromosome. In our experiments, CDX males inherited Y chromosomes that had had the opportunity to evolve in male-limited X populations, along with paternally-transmitted X chromosomes. As Table 2 shows, these males display broad-scale up-regulation of X-linked loci, instead of the feminized X-expression patterns predicted by our imprinting hypothesis. It is conceivable that as male-benefit sexually antagonistic alleles become enriched on the X-chromosome – making the X-chromosome more “masculine” – there is coincident selection for epistatic up-regulatory effects of the Y-chromosome. This is not a far-fetched suggestion, as the Y is highly heterochromatized, polymorphic, and is known to broadly influence gene expression levels [40], [50], [51], [53]. This would suggest that Y-chromosome variants in normal fly populations exist that are favoured in combination with feminized X-chromosomes. Such coevolution would be unique to our experiment, as previous did not allow for coevolution of X and Y chromosomes [8], [43], [46]. Experiments are currently in preparation which will directly address this evolutionary explanation for rapid evolution and diversification of the Y-chromosome.

In summary, we found no clear evidence that male imprinting of the X-chromosome helps to resolve intralocus sexual conflict in *Drosophila melanogaster*. This conclusion must necessarily be considered tentative at this point, since some of our transcript categories could not unambiguously disentangle possible imprinting effects from other sorts of maternal effects or from the response to MLX evolution. The mechanism causing the deleterious maternal effects seen in DX females is also unknown, so it is not impossible that abnormal imprinting of the Y-chromosomes or autosomes by DX females is the proximate cause. The speed of resolution of intralocus sexual conflict has been a matter of some discussion [3], [4], [10], and different mechanisms are likely to work on different time scales. Recombination and fixation of standing genetic variation or sex-specific maternal effects will likely produce the fastest response, while the evolution of sex-specific modifiers or relocation of sexually antagonistic genes to the sex chromosomes should work on longer time scales [3], [4], [10]. Genomic imprinting may therefore act on different time scales in different species depending on the imprinting mechanism and genomic architecture. Changes in the gene pool of Y-chromosomes driven by epistatic interactions or Y-linked imprinting effects may also explain why we could not detect a signal of evolution in specifically X-linked imprinting effects. However our data suggest that the evolution of maternal effects are likely to contribute to the resolution of intralocus sexual conflict, and confirm at the level of the transcriptome that the X-chromosome is a hotspot for sexually antagonistic fitness variation. They also suggest that further study of the potential for coevolution of the X- and Y-chromosomes is warranted, and that caution should be used when interpreting the results of assays which use chromosomal tools such as attached X-chromosomes.

## Supporting Information

File S1 **Tables S1–S3 & Figures S1–S4.**
(DOCX)
