# Supplementary material for: Epigenetics and Sex-Specific Fitness: An Experimental Test Using Male-Limited Evolution in Drosophila melanogaster
Source: PLoS One. 2013 Jul 29;8(7):e70493. doi: 10.1371/journal.pone.0070493 (PMC3726629; doi:10.1371/journal.pone.0070493)
Supplement: File S1 — Tables S1–S3 & Figures S1–S4. (DOCX) [file pone.0070493.s001.docx]

Epigenetics and sex-specific fitness: an experimental test using male-limited evolution in *Drosophila melanogaster,* Supplemental information

Jessica K. Abbott^1,2^, Paolo Innocenti^3^, Adam K. Chippindale^4^, & Edward H. Morrow^5^

1. Corresponding author

2. Department of Biology

Section for Evolutionary Ecology

Lund University

Sölvegatan 37

22362 Lund, Sweden

3. Department of Animal Ecology

Evolutionary Biology Centre

Uppsala University

Norbyvägen 18D

SE-75236 Uppsala, Sweden

4. Biology Department

Queen’s University

Kingston, Ontario, Canada

K7L 3N6

5. School of Life Sciences

University of Sussex

John Maynard Smith Building

Brighton, UK

BN1 9QG

Table S1: Overrepresented Gene Ontology (GO) terms for “MLX evolution” transcripts (i.e. those that were significantly different in the MLX treatment compared to the CDX and Control treatments).

| Domain | ID | P-value | Odds ratio | Expected count | Observed count | Size | Term |
| --- | --- | --- | --- | --- | --- | --- | --- |
| Biological process | GO:0030241 | 0.000 | 318.167 | 0 | 2 | 3 | Skeletal muscle thick filament assembly |
| Biological process | GO:0031033 | 0.000 | 318.167 | 0 | 2 | 3 | Myosin filament assembly or disassembly |
| Biological process | GO:0031034 | 0.000 | 318.167 | 0 | 2 | 3 | Myosin filament assembly |
| Biological process | GO:0014866 | 0.000 | 106.000 | 0 | 2 | 5 | Skeletal myofibril assembly |
| Biological process | GO:0030239 | 0.003 | 28.848 | 0 | 2 | 13 | Myofibril assembly |
| Cellular component | GO:0005615 | 0.001 | 19.857 | 0 | 3 | 26 | Extracellular space |
| Cellular component | GO:0005576 | 0.002 | 5.286 | 1 | 6 | 197 | Extracellular region |
| Cellular component | GO:0030016 | 0.004 | 26.521 | 0 | 2 | 13 | Myofibril |
| Cellular component | GO:0030017 | 0.004 | 26.521 | 0 | 2 | 13 | Sarcomere |
| Cellular component | GO:0044449 | 0.005 | 22.427 | 0 | 2 | 15 | Contractile fiber part |
| Cellular component | GO:0044421 | 0.007 | 9.057 | 0 | 3 | 53 | Extracellular region part |
| Cellular component | GO:0043292 | 0.007 | 19.424 | 0 | 2 | 17 | Contractile fiber |
| Cellular component | GO:0005778 | 0.007 | Inf | 0 | 1 | 1 | Peroxisomal membrane |
| Cellular component | GO:0016942 | 0.007 | Inf | 0 | 1 | 1 | Insulin-like growth factor binding protein complex |
| Cellular component | GO:0031903 | 0.007 | Inf | 0 | 1 | 1 | Microbody membrane |
| Cellular component | GO:0044438 | 0.007 | Inf | 0 | 1 | 1 | Microbody part |
| Cellular component | GO:0044439 | 0.007 | Inf | 0 | 1 | 1 | Peroxisomal part |
| Molecular function | GO:0004097 | 0.007 | Inf | 0 | 1 | 1 | Catechol oxidase activity |
| Molecular function | GO:0004332 | 0.007 | Inf | 0 | 1 | 1 | Fructose-bisphosphate aldolase activity |
| Molecular function | GO:0004503 | 0.007 | Inf | 0 | 1 | 1 | Monophenol monooxygenase activity |
| Molecular function | GO:0008288 | 0.007 | Inf | 0 | 1 | 1 | Boss receptor activity |
| Molecular function | GO:0016682 | 0.007 | Inf | 0 | 1 | 1 | Oxidoreductase activity, acting on diphenols and related substances as donors, oxygen as acceptor |
| Molecular function | GO:0016716 | 0.007 | Inf | 0 | 1 | 1 | Oxidoreductase activity, acting on paired donors, with incorporation or reduction of molecular oxygen, another compound as one donor, and incorporation of one atom of oxygen |

Table S2: Overrepresented Gene Ontology (GO) terms for “fitness” transcripts (i.e. those that were up- or down-regulated in the order CDX-C-MLX).

| Domain | ID | P-value | Odds ratio | Expected count | Observed count | Size | Term |
| --- | --- | --- | --- | --- | --- | --- | --- |
| Biological process | GO:0035202 | 0.005 | Inf | 0 | 1 | 1 | Sac formation, open tracheal system |
| Biological process | GO:0006508 | 0.006 | 5.365 | 1 | 5 | 261 | Proteolysis |
| Biological process | GO:0007174 | 0.009 | 225.059 | 0 | 1 | 2 | Epidermal growth factor ligand processing |
| Biological process | GO:0007176 | 0.009 | 225.059 | 0 | 1 | 2 | Regulation of epidermal growth factor receptor activity |
| Biological process | GO:0007508 | 0.009 | 225.059 | 0 | 1 | 2 | Larval heart development |
| Biological process | GO:0010469 | 0.009 | 225.059 | 0 | 1 | 2 | Regulation of receptor activity |
| Biological process | GO:0035311 | 0.009 | 225.059 | 0 | 1 | 2 | Wing cell fate specification |
| Cellular component | GO:0005792 | 0.001 | 20.993 | 0 | 3 | 41 | Microsome |
| Cellular component | GO:0042598 | 0.001 | 20.993 | 0 | 3 | 41 | Vesicular fraction |
| Cellular component | GO:0016020 | 0.001 | 5.924 | 4 | 10 | 825 | Membrane |
| Cellular component | GO:0005624 | 0.001 | 17.689 | 0 | 3 | 48 | Membrane fraction |
| Cellular component | GO:0000267 | 0.001 | 16.926 | 0 | 3 | 50 | Cell fraction |
| Cellular component | GO:0005626 | 0.001 | 16.926 | 0 | 3 | 50 | Insoluble fraction |
| Molecular function | GO:0004497 | 0.004 | 10.979 | 0 | 3 | 58 | Monooxygenase activity |
| Molecular function | GO:0009055 | 0.005 | 9.724 | 0 | 3 | 65 | Electron carrier activity |
| Molecular function | GO:0020037 | 0.005 | 9.724 | 0 | 3 | 65 | Heme binding |
| Molecular function | GO:0046906 | 0.005 | 9.724 | 0 | 3 | 65 | Tetrapyrrole binding |
| Molecular function | GO:0005332 | 0.006 | Inf | 0 | 1 | 1 | Gamma-aminobutyric acid:sodium symporter activity |
| Molecular function | GO:0008233 | 0.006 | 4.954 | 1 | 5 | 221 | Peptidase activity |

Table S3: Results of test for feminization of autosomal transcripts in CDX males. If CDX males (i.e. males with a paternally transmitted X-chromosome, produced by crossing a Control male to a DX female) have feminized expression of autosomal transcript due to imprinting of the X-chromosome, then the change in expression of autosomal transcripts relative to Control males should be in the same direction as extant sexual dimorphism more often than expected by chance (first and last columns). The transcripts are distributed among the categories significantly non-randomly (χ^2^ = 707.6135, df = 3, *P*-value < 2.2*10^-16^) but the observed pattern is not consistent with feminization, similar to the results from the X-linked transcripts (Table 1 in the main text). Note that the total number of transcripts in the analysis is less than the total number of genes because uninformative transcripts (i.e. those without gene annotation information, or those whose expression was the same across all samples) were filtered out during pre-processing.

|  | Up-regulated in females | | Down-regulated in females | |
| --- | --- | --- | --- | --- |
|  | **Up-regulated in CDX** | **Down-regulated in CDX** | **Up-regulated in CDX** | **Down-regulated in CDX** |
| Observed | 570.00 | 395.00 | 1366.00 | 714.00 |
| Expected | 761.25 | 761.25 | 761.25 | 761.25 |


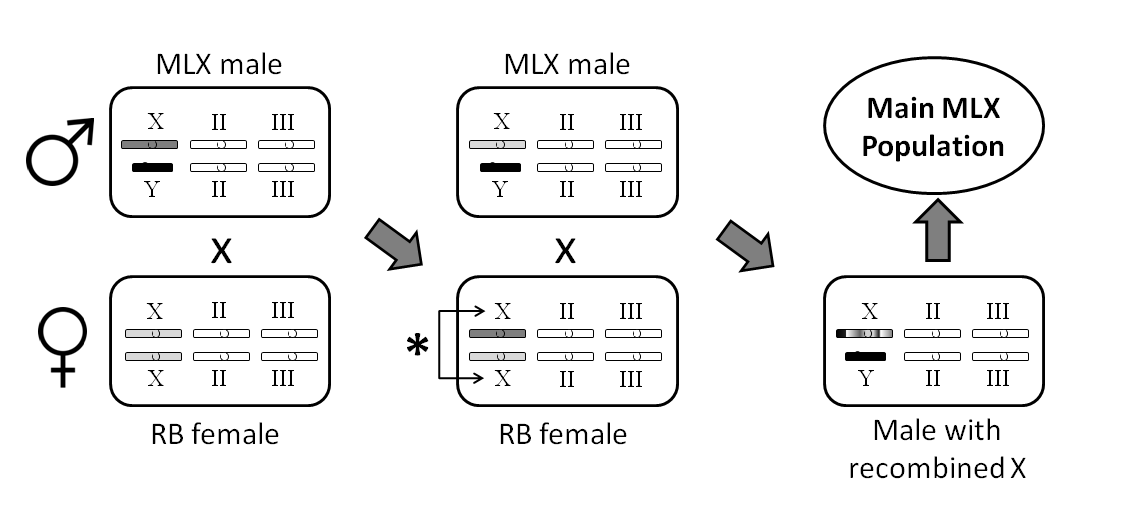


Figure S1: MLX recombination box protocol. An MLX male harvested from the main MLX population is mated to an RB female, producing RB daughters with a paternally inherited ML X-chromosome (dark grey) and a maternally inherited X-chromosome (light grey). These two X-chromosomes are then recombined in the female during egg production (asterisk), resulting in the production of sons with a recombined X-chromosome (marbled grey). This results in a constant inflow of unrecombined X-chromosomes to the RB population, and a constant outflow of recombined X-chromosomes from the RB population back to the main MLX population.

**Production of MLX and CDX males for assays:**


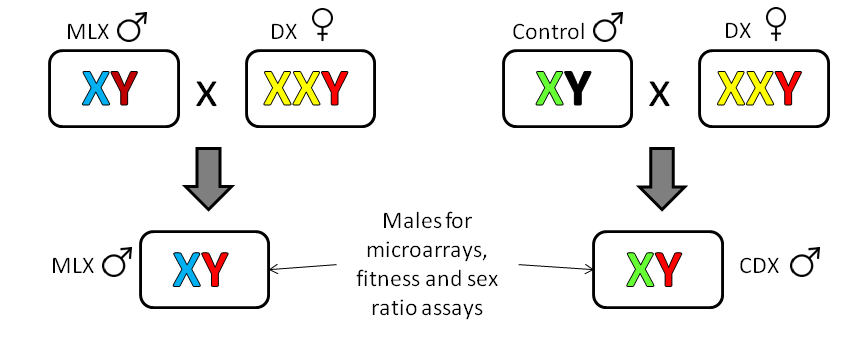


**MLX and CDX sex ratio assays:**


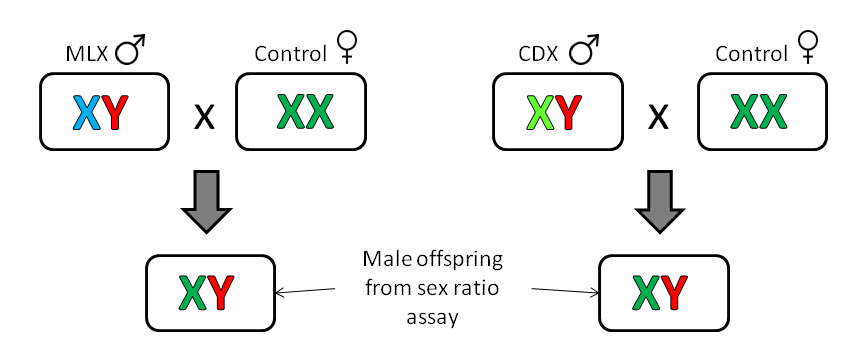


Figure S2: Production of males for MLX and CDX experimental treatments (top) and sex ratio assay protocols (bottom). MLX-derived X-chromosomes are indicated in blue, Control-derived X-chromosomes in shades of green. MLX-derived Y-chromosomes are indicated in shades of red, Control-derived Y-chromosomes in black. The DX construct is indicated in yellow. Note that the differences in offspring sex ratio between MLX and CDX males cannot be due X-linked or Y-linked genetic effects in isolation, since all male offspring have a Control-derived X-chromosome and an MLX-derived Y-chromosome. Rather, it seems that male offspring survival is determined by whether or not the paternal sex chromosomes are mismatched (see Figure S3).

Figure S3: Patterns of significant differences between treatments. The yellow circle (yellow, green, orange, and white areas) represents transcripts that differed significantly between the Control and CDX males. These transcripts are likely to encompass epigenetic effects. The red circle (red, purple, orange, and white areas) represents transcripts that differed significantly between the MLX and CDX males. These transcripts are likely to encompass effects of MLX evolution. The blue circle (blue, purple, green, and white areas) represents transcripts that differed significantly between the Control and MLX treatments. For these transcripts we cannot distinguish between effects of MLX evolution and maternal effects of the DX females. When ignoring the ambiguous blue area, it is clear that effects of MLX evolution (red circle) seem to be more common than epigenetic effects (yellow circle).

Note that for the most part, the various coloured areas correspond well to the expression categories in Figure 2 of the main text. Green = Category 1 (maternal effects of DX females; MLX and CDX significantly different from Control, but not significantly different from each other). Orange = Category 2 (imprinting effects; CDX significantly different from MLX and Control, but no difference between MLX and Control). Purple = Category 3 (effects of MLX evolution; MLX significantly different from both CDX and Control). Red = Category 4 (fitness-related transcripts; rank-order CDX-C-MLX). Yellow = Category 5 (deleterious maternal effects; rank-order CDX-MLX-C). Blue = Category 6 (beneficial maternal effects; rank-order C-CDX-MLX). Of the 6 transcripts in the white area (all differences significant), one was assigned to Category 5 and five to Category 4. Note that five transcripts from Category 5 appear in the blue area rather than in the yellow area. This is because these particular transcripts had a rank-order CDX-MLX-C, but the standard errors for the CDX treatment were large enough that the CDX-MLX and CDX-C comparisons were marginally non-significant, and only the MLX-C comparison significant. This small discrepancy between the two methods of classification of transcripts (Categories vs. pairwise differences) has no effect on our qualitative conclusions since we have treated both groups as types of maternal effects.


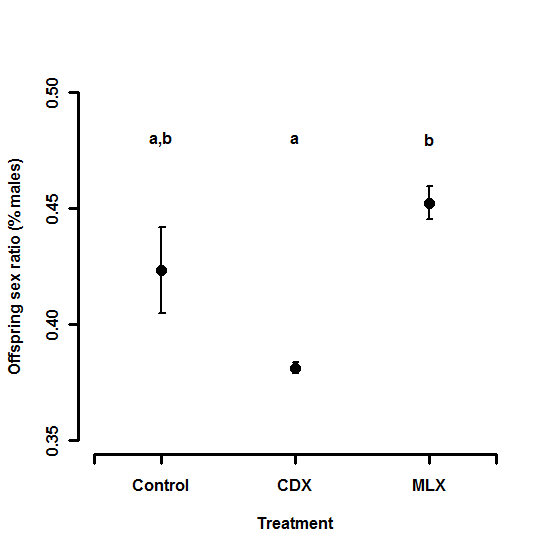


Figure S4: Differences in adult offspring sex ratio according to treatment. MLX males have significantly higher numbers of adult male offspring than CDX males. Note that the slight overall female bias is normal for the LHm source population.
